# Prevention of re-establishment of malaria: historical perspective and future prospects

**DOI:** 10.1186/s12936-020-03527-8

**Published:** 2020-12-07

**Authors:** S. M. Ibraheem Nasir, Sachini Amarasekara, Renu Wickremasinghe, Deepika Fernando, Preethi Udagama

**Affiliations:** 1 Department of Zoology & Environment Sciences, Faculty of Science, University of Colombo, Colombo 3, Sri Lanka; 2 Department of Parasitology, Faculty of Medical Sciences, University of Sri Jayewardenepura, Nugegoda, Sri Lanka; 3 Department of Parasitology, Faculty of Medicine, University of Colombo, Colombo 8, Sri Lanka

**Keywords:** Malaria elimination, *Plasmodium vivax*, *Plasmodium falciparum*, Prevention of re-establishment, Importation risk, Surveillance, Receptivity, Vulnerability

## Abstract

Prevention of re-establishment (POR) refers to the prevention of malaria outbreak/epidemic occurrence or preventing re-establishment of indigenous malaria in a malaria-free country. Understanding the effectiveness of the various strategies used for POR is, therefore, of vital importance to countries certified as “malaria-free” or to the countries to be thus certified in the near future. This review is based on extensive review of literature on both the POR strategies and elimination schemes of countries, (i) that have reached malaria-free status (e.g. Armenia, Mauritius, Sri Lanka), (ii) those that are reaching pre-elimination stage (e.g. South Korea), and (iii) countries at the control phase (e.g. India). History has clearly shown that poorly implemented POR programmes can result in deadly consequences (e.g. Sri Lanka); conversely, there are examples of robust POR programmes that have sustained malaria free status that can serve as examples to countries working toward elimination. Countries awaiting malaria elimination status should pre-plan their POR strategies. Malaria-free countries face the risk of resurgence mostly due to imported malaria cases; thus, a robust passenger screening programme and cross border collaborations are crucial in a POR setting. In addition, sustained vigilance, and continued funding for the national anti-malarial campaign programme and for related research is of vital importance for POR. With distinct intrinsic potential for malaria in each country, tailor-made POR programmes are built through continuous and robust epidemiological and entomological surveillance, particularly in countries such as Sri Lanka with increased receptivity and vulnerability for malaria transmission. In summary, across all five countries under scrutiny, common strengths of the POR programmes are (i) a multipronged approach, (ii) strong passive, active, and activated passive case detection, (iii) Indoor residual spraying (IRS), and (iv) health education/awareness programmes.

## Background

Malaria is a life-threatening disease that has ravaged human lives for thousands of years; 228 million cases of malaria accompanied by 405,000 deaths were reported globally in 2018 [1]. Over 90% of these cases and the deaths were reported from the African region, and 67% of the global malaria deaths were children under the age of 5 years [1]. Of the five parasite species that cause malaria in humans, *Plasmodium vivax* and *Plasmodium falciparum*, approximately in the same proportion, pose the greatest threat being accountable for the majority of the estimated malaria cases worldwide [2].

Interventions to reduce the prevalence of malaria were expanded drastically, reducing the incidence of the population at risk from 71 to 57 cases per 1000 from 2010–2018 [1]. However, a significant reduction of percentage of people at risk was only visible in the South-east Asia region (70% decrease), whereas in the Americas a rise in the incidence was reported [1]. A reduction of 22% of people at-risk was also reported in the World Health Organization (WHO) Africa region from the year 2010 to 2018. An overall reduction in the malaria mortality rate was reported in 2018 in comparison to 2010 in WHO Africa (from 533 000 to 380 000) and South-East Asia (from 39000to 12,000) regions. However, the reduction rate of malaria mortality has lowered since 2016, indicating the trends of malaria case incidence and gaps of malaria control strategies [1].

The WHO is at the forefront of the fight against malaria and expecting to reduce the incidence of malaria by 90% by 2030, reduce the rate of mortality due to malaria by 90% by 2030, to reach elimination in at least 35 countries by 2030 and to prevent re-establishment of malaria in all malaria-free countries [3]. WHO certification for malaria elimination is awarded to countries that report zero incidence of indigenous malaria cases, through three consecutive years. Between 1955 to 2019, 38 countries were awarded malaria-free certification including the most recent awardees—Morocco (2010), Turkmenistan (2010), Armenia (2011), Maldives (2015), Sri Lanka (2016), Kyrgyzstan (2016), Paraguay (2018), Uzbekistan (2018), Algeria (2019) and Argentina (2019) (Table 1).

**Table 1:** Countries and territories certified malaria free by WHO from 1973 and their prevention of re-establishment (POR) strategies

Country	WHO certificate of elimination	Techniques implemented within POR programme
Mauritius	1973	Strong case detection programme (PCD/ACD/RACD), Integrated Vector Management (IVM), a strong healthcare system that responds promptly to newly introduced cases, IRS at ports of entry, prophylaxis for travellers, surveillance of incoming passengers, education about malaria and information for medical personnel on malaria case management [4–7]
Australia	1981	Joining Australian Government, State and Territory initiative through Australian Government Arbovirus and Malaria Surveillance Website to assist health authorities in the reduction of malaria by rapid and greater dissemination of national malaria surveillance data, transfer of interpretations of these data and support information between health authorities, aiding early recognition of unusual mosquito and malaria activity provide access for the general public to malaria surveillance information, and increasing public awareness of the potential mosquito-borne disease risks in their region. Pre-arrival assessment, screening and appropriate treatment of all refugees [8, 9]
La Reunion (France)	1979	Permanent, optimized epidemiologic and entomological surveillance and vector control measures, imported and local malaria cases, detection and control of malaria cases; targeted anti-vector activity based on a systematic anti-larval control, eventually completed by the eradication of the adapted adult vectors [10, 11]
Singapore	1982	Surveillance and control of *Anopheles* mosquitoes and identification of specific malaria receptive areas, case surveillance and epidemiological investigation, compulsory malaria screening for foreign workers from 1997 as part of the pre-employment medical examinations, early case detection through blood and fever surveys in malaria receptive areas and risk communication to medical practitioners as well as health education for the public [12, 13]
Brunei Darussalam	1987	Vector Control, Entomology and Malaria Vigilant Unit is committed in carrying out activities to prevent the indigenous transmission and re-establishment of malaria into the country, malaria vigilance unit- prevention of reappear or re-establishment of malaria into the free status country, entomology unit-monitors the prevalence, distribution and density of public health important vectors, vector control unit [14]
UAE	2007	Immediate notification of all imported cases of malaria, free diagnosis and treatment for patients, including the significant percentage of travellers to the country. Continuous case detection programmes and public awareness campaigns to achieve community cooperation are also important parts of the strategy. The malaria programme was also recommended to be integrated with the health care system (IVM) [55]
Morocco	2010	National Malaria Control Programme has a considerable inventory of entomological and parasitological information, and several areas of high risk are regularly studied and monitored [15, 16]
Turkmenistan	2010	Maintenance of epidemiological surveillance of malaria to ensure prompt detection and treatment of cases and timely response to any emergency. Prompt and timely response to changes in the receptivity and vulnerability of the territory of the country [17, 18]
Armenia	2011	Adaptation of the epidemiological surveillance system to match with POR, establishment of a cross-border cooperation policy, improved preventive and anti-epidemic measures in foci of infection, preventive activities and measures for high-risk groups, dissemination of information on malaria prevention and hygiene to the population, and recruitment and training personnel for malaria prevention [17]
Maldives	2015	Epidemiological surveillance by the health care providers and the community, prevention through port health and international travel health, effective health care, integrated vector surveillance and control [19]
Sri Lanka	2016	Focused strongly on vector control, educating health personnel and the public on the risk of POR, strong surveillance methods for the treatment of imported cases [55]. After elimination of malaria, active case detection with mobile malaria units and passive case detection is still maintained and vigilantly diagnosing every imported case of malaria, promptly treating the cases, incorporated with radical cure with primaquine [30]
Kyrgyzstan	2016	Early diagnosis and notification of all cases of malaria and timely radical treatment, identification of all cases and causes of any re-establishment of malaria transmission, immediate response in case of re-establishment of transmission, continuous training and retraining of health care professionals, increased social mobilization and coordinated intersectoral actions, partnerships with international and donor organizations and cross-border cooperation [17]
Paraguay	2018	Trainings across general health services to maintain vigilance and ongoing engagement with community volunteers, which ensures the prompt detection and treatment of cases. Monitor changes related to the risk of imported malaria by collaborating authorities in the national malaria programme across sectors and ministries [20]
Uzbekistan	2018	Maintenance of malaria surveillance to detect malaria cases rapidly and take necessary action, monitoring persisting levels of receptivity and vulnerability, early case detection, with special attention to identifying imported cases by vigilant surveillance, a competent general health service and strong support from quality assurance laboratories, and a strong information system, with obligatory notification and reporting of malaria and timely epidemiological investigation of each case and focus [17]
Argentina	2019	Integration of malaria surveillance into the national surveillance system, which allows suspected malaria cases to be rapidly identified and tested. Integration of malaria prevention and treatment services into a primary health care system that engages a large cadre of paid community health workers in the areas where the risk of re-establishment of malaria is high. By integrating malaria into the system they respond to cholera and dengue outbreaks that will enable quick interruption of transmission of malaria [21]
Algeria	2019	Identification of malaria cases by trained personnel, vector surveillance, and effective oversight by provincial and national health experts to ensure that any local and imported cases are quickly identified, and that appropriate actions are taken to prevent re-establishment of transmission [22]

Subsequent to being malaria-free, the occurrence of three or more indigenous cases of the same species per year in the same focus for three consecutive years results in acquiring “Re-establishment of transmission” status [23, 24]. The risk of re-establishment is primarily through “imported” malaria cases where the infection is acquired outside the geographic area in which it is diagnosed (visitors and migrants from endemic regions) and *Anopheles* mosquitoes can resume the spread of the disease in favourable environmental conditions [5].

Prevention of re-establishment (POR) is the prevention of malaria outbreak/epidemic occurrence or preventing re-establishment (occurrence of 3 or more indigenous cases of the same species per year in the same focus for 3 consecutive years) of indigenous malaria in a malaria-free country [5, 6]. Considering the past malaria epidemic patterns, the risk of re-establishment is plausible in malaria-free countries that do not have adequate surveillance systems, adequate malaria vigilance and skilled personnel for diagnosis and treatment. Therefore, POR of malaria is an important step in countries with malaria-free certification. This review comprehends the strategies used by countries to successfully sustain or achieve the “malaria free status”, and to highlight lessons learnt by those who suffered from the re-establishment of malaria transmission.

### Intrinsic potential for malaria

For countries that have eliminated the disease or are at the pre-elimination stage, vigilant surveillance must be in place to prevent re-establishment of transmission. Re-establishment is achieved through high vulnerability and high receptivity, and mainly by the importation of a parasite to a receptive area with a susceptible human population leading to a local infection. Importation risk or the vulnerability of malaria is the rate of influx of infected individuals and/or infective *Anopheles* mosquitoes [7]. Receptivity of the ecosystem relies on the competency of vectors, vector density and longevity, suitable climate and a susceptible population [25, 26]. Therefore, the intrinsic potential for malaria transmission is highly dependent on environmental and socio-economic factors [27, 28]. Tropical countries thus have a high receptivity for malaria transmission that can make the POR comparatively challenging [27, 28]. Basic reproduction number (R_0_) plays an important role in malaria epidemiology and other infectious diseases as it leads to the understanding of transmission intensity [27]. R_0_ is the number of individual hosts that are to be infected when an infected host is introduced into a naïve population, after one reproductive cycle [27]_._ If in a population R_0_ is greater than one, the number of infected individuals will increase whereas, if R_0_ is less than one, the number of infected individuals would decrease. Thus, if disease control is in place, low transmission intensity (R_0_ < 1) will lead to the eventual elimination of the parasite.

### History of malaria elimination and POR

Surveillance is an important component of any POR setting, which includes a continuous, systemic collection, analysis, and interpretation of data that will help the country to monitor and evaluate the disease emerging patterns and trends. Passive case detection (PCD), proactive/active case detection (ACD), and reactive/activated passive case detection (RACD) are various forms of surveillance methods used in malaria elimination/POR. PCD refers to the detection triggered by the patients presenting at the health clinics for malaria-like symptoms followed by notifying it to the epidemiological surveillance systems [29]. ACD refers to the screening of high-risk populations such as migrants by staff reaching out to the community [30]. RACD refers to the ACD that is restricted to the closest areas of passively detected cases; this has been practiced in countries such as South Africa, Swaziland, Brazil, Zambia, Peru, and Swaziland for effective malaria control [30]. Gaps in surveillance, prevention or funding have caused disastrous recurrences of malaria in the past. Lately, China has successfully implemented the 1–3-7 strategy for malaria control and it includes reporting of any malaria case within day 1, confirmation and investigation of the case within day 3, and taking measures to ensure no further spread by the end of day 7 [31].

In 1955, the WHO launched the Global Malaria Eradication Programme (GMEP), the first global health programme aimed at “total coverage” with the approval of the World Health Assembly [32]. To reach a higher goal of eradication, indoor residual spraying (IRS) with dichlorodiphenyltrichloroethane (DDT) or other approved insecticides was recommended abandoning previous measures, such as demolishing of mosquito breeding spots, prevention of mosquito bites, and other traditional malaria control methods [33]. GMEP contributed positively on reducing the geographical distribution of malaria, with a significant influence on health programme planning, establishing community networks through voluntary collaborators for diagnosis and treatment and creating of surveillance systems capable of detecting the last cases of malaria [33, 34]. However, during GMEP social and cultural barriers were disregarded, complementary measures such as usage of anti-malarial drugs were considered non-essential and no recognition was given for country programmes to provide a useful contribution [35]. Sociocultural factors such as poverty and social inequalities put people at continuous risk of malaria. Underprivileged communities with poor housing were more exposed to mosquitoes and with poor nutrition; they became more vulnerable to malaria or any other disease. Poverty also contributed to poor sanitation, low medical attention, lack of education/knowledge increasing the risk of malaria resurgence after eliminating malaria by a chronic anti-malarial intervention [35]. Failing to advance as expected and resurgence in some areas were observed in the 1960s and the WHO had reduced capacity to fund GMEP [33, 36]. In 1967, the re-examination of the global strategy for malaria was probably due to the resurgence of malaria in Sri Lanka [37]. In 1969, identifying that eradication was not achievable with the available means in certain areas, GMEP was discontinued. Lessons learnt from GMEP (1955–1969) revealed that no single strategy can be applied in malaria prevention/POR and that a continuous commitment, an active surveillance system that do not disregard the social and cultural barriers of a country developed with the involvement of the community, epidemiologists, entomologists, researchers, and other personnel affiliated to health systems is critically needed [33].

Hard lessons can be learnt specifically by observing a country that suffered a particularly vicious case of malaria re-establishment in the past, such as Sri Lanka, which is detailed later on in this review. Since the year 2000, the number of successful interventions to combat malaria transmission has increased globally due to the increased flow of funding into these programmes. As a result, the prevalence of malaria even in highly endemic regions of the world has declined [38]. These positive outcomes undoubtedly show that well planned anti-malaria campaigns with sustained funding have an enormous impact on the global malaria burden. However, the GMEP history highlights that even the slightest misstep can reverse all strides made to eliminate malaria [33].

### Causes and prevention of re-establishment

One of the major causes for re-establishment of malaria in a country is the reduction of funding for anti-malaria programmes following elimination, burdening the government to invest in a full-scale malaria prevention campaign. In the late 1950s, the United States Agency for International Development (USAID) provided DDT to India leading to a significant reduction in the prevalence of malaria, from an estimated 100 million annual cases in the early twentieth century to about 100 thousand cases in 1965 [39]. However, reduction of malaria incidence in India lead USAID to withdraw its funding, expecting the Indian government to shoulder the financial costs; but India could not manage to produce or procure the large amount of DDT required. The lack of DDT was likely a key element in the subsequent re-establishment of malaria to a peak of 6 million cases by 1976 [39]. Lack of malaria vigilance is also another reason that can lead to re-establishment [40]. The WHO has revealed that Mauritius upon elimination of malaria in 1969 has had lax control measures, malfunctioning anti-malarial programmes, scaled back IRS and larviciding schedules and offhand regular malaria testing, that subsequently led to the re-establishment of malaria in 1975 [7]. In addition, the WHO has highlighted on the lack of international information available on the status of malaria in different regions, lack of information provided to the people leaving the region by the malarious countries, challenges in establishing checked passage of people crossing the borders, inadequate awareness about the possibilities of re-establishment to malaria-free regions, in people/health sector staff and lack of malaria training as causes of re-establishment [40]. Moreover, drug and insecticide resistance, natural disasters and war that can augment the intrinsic potential for malaria may increase the potential of re-establishment [28].

**Fig. 1:**
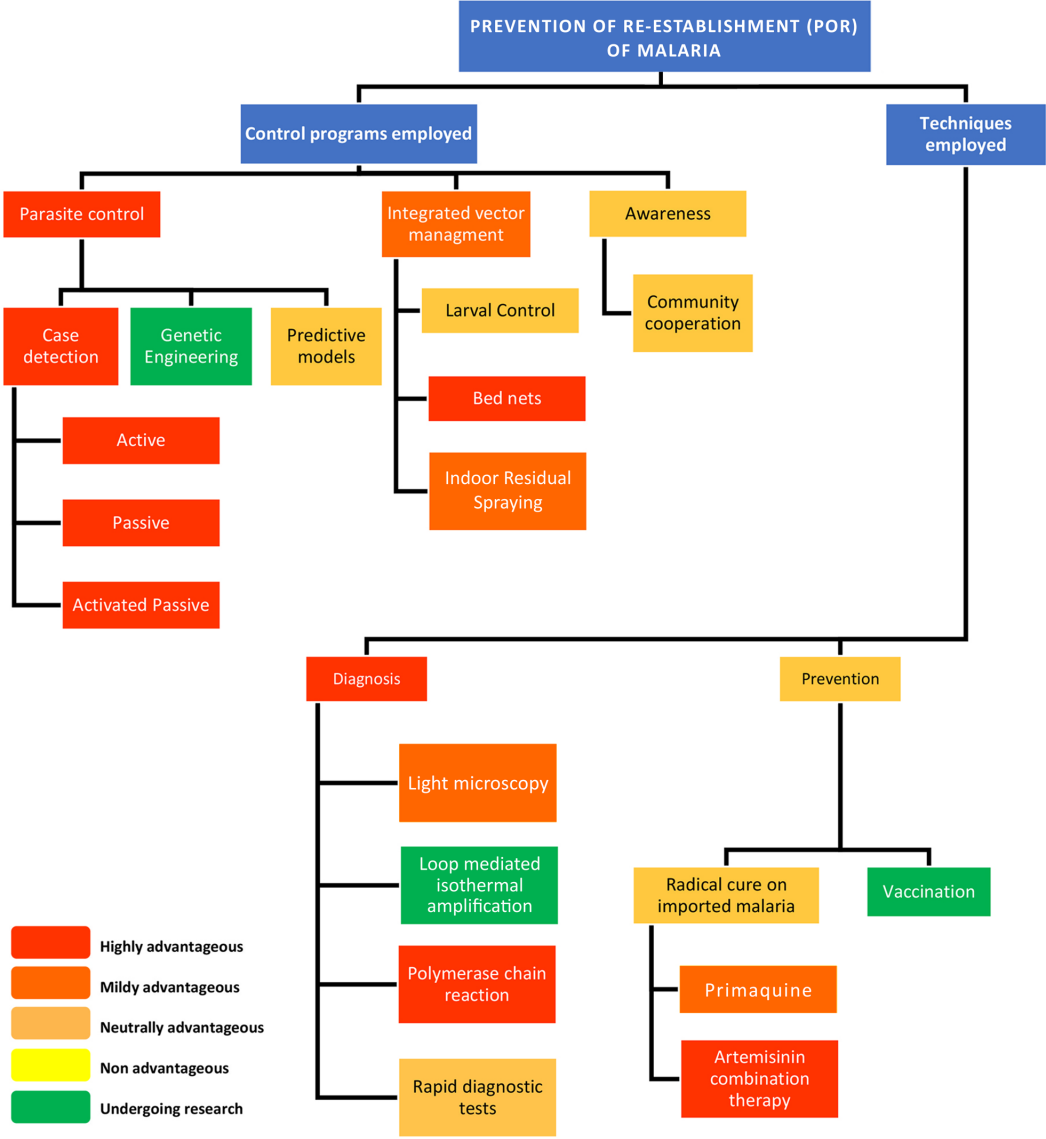
Potential techniques to be used during the prevention of malaria re-establishment phase

A variety of programmes/techniques has been recognized as ideal methods to be used for the POR of malaria (Fig. 1). While the global scope of POR is reviewed herein, it is important to note that each country has its specific outbreak and importation risk, and a POR programme must be tailor-made to address the specific risks of a country focussed on reducing the malariogenic potential. However, the WHO has placed the following essential guidelines for POR; (i) implementing an efficient malaria surveillance system, (ii) keeping the POR budget at least at the same level of pre-elimination, (iii) continuation of staff education/training programmes for general health services personnel to diagnose, treat and being vigilant, (iv) training freshers in malaria diagnosis and treatment, (v) establishing or maintaining country/ region/sub-region specific malaria prevention services according to the epidemiological data sources [40]. Development of a national malaria prevention plan in collaboration with entomologists and epidemiologists particularly with the activities directed against malaria vectors is also of vital importance [40]. Moreover, general health services authorities hold the responsibilities of detecting all suspected local and migrant malaria cases, notification of such cases and epidemiological investigations, and administration of medication to each patient [40].

## Methods

An extensive internet search was performed, for scientific publications indexed in the PubMed/Medline database using the following keywords; *P. vivax*, *P. falciparum,* malaria elimination, prevention of re-establishment, importation risk, surveillance. Measures oriented towards gaining/ sustaining malaria-free status by different countries were specifically focused upon.

In this study, three countries at the POR phase were included: Armenia, Mauritius and Sri Lanka (Table 2). Availability of information on POR of malaria control programmes was a contributory factor in selecting these countries. India was selected for its population size and as a country failing to achieve complete elimination countless times, despite best efforts. South Korea was chosen for the lessons that can be learnt from a country that has experienced re-establishment.

**Table 2:** Population, geography and malaria history of countries selected for this review

Country	Population	Geography	Current phase
Sri Lanka	20,966,000	Island	Prevention of re-establishment phase
Armenia	3,017,712	Continental	Prevention of re-establishment phase
Mauritius	1,262,605	Island	Prevention of re-establishment phase
South Korea	51,470,000	Continental	Entering pre-elimination
India	1,311,000,000	Continental	Control phase

Data Set: World Bank: SP.POP.TOTL

## Findings

### Mauritius

Mauritius is a cautionary tale for countries at the stage of POR of malaria. Malaria was introduced to Mauritius in the 1860s and became endemic in the country by 1946 [41, 42]. From 1948 to 1952, IRS with DDT was carried out according to GMEP recommendations leading to a drop in cases of malaria from 105 to 2.6 per 1000 population [43]. Moreover, targeted spraying, fever surveys, and a strong surveillance system consisting of a mobile malaria squad lead Mauritius to receive malaria-free status in 1973. During the first POR stage in Mauritius, routine island-wide larviciding and DDT spraying at the ports of entry initially every three (1968–1970) or six (1971–1974) months was carried out to control the vectors [44–48]. The POR programme was also supported by robust passenger screening to prevent importation and fever surveys [44, 45].

Yet, in 1975, *P. vivax* was re-established in the country, spurred by a devastating cyclone that allowed breeding grounds to develop and the migration of workers from malaria-endemic India to help rebuild [49]. Negligent surveillance interventions after elimination of malaria, absence of passive surveillance methods, and increased importation risk from the significant influx of migrant workers/visitors was potentially attributable to re-emergence of malaria in the country [7]. With 623 indigenous cases, the malaria epidemic peaked in 1982. However, by 1998 Mauritius became malaria-free by usage of IRS, widespread larviciding, passenger screening, and an extensive case response system [50, 51]. Since then, the country has once again achieved elimination which has been sustained since 1998 [49]. Although the WHO has provided limited financial support with other resources, such as instruments and insecticides, the national government remained to be the principal funding body in achieving this goal and the Mauritius government currently spends $2.06 per capita per year on its POR programme [4, 7]. The current POR programme in Mauritius consists of the following three principal strategies diligently practiced at present, which however were also used in previous programmes:

1. Continuation of the PCD/ACD/RACD surveillance programme in people travelling from malaria-endemic countries, report having been in a malaria-endemic country in the last six months, and/or those who report being febrile upon arrival [5]. Prior to November 2008, blood films were made for A(CD from all passengers complaining of fever; however, currently the febrile passengers are directed to public or private hospitals near the port of entry [7].
2. Integrated Vector Management (IVM) strategy: IVM is a strategy recommended by the WHO to be utilized during the POR phase. It is described as a rational decision-making process to optimize the use of resources for vector control. This includes evidence-based decision-making, integrating with other vector control programmes, development of legislation, advocacy and awareness, and capacity building [6]. The techniques employed in this strategy are continuous with IRS and larviciding in previously endemic areas and at ports of entry [4].
3. A strong healthcare system that responds instantly to newly introduced malaria cases [7].

### Armenia

Armenia suffered a serious malaria epidemic during the 1920s to the 1930s but from the 1940s to the 1950s, the situation improved rapidly as the country invested in the development of a malaria control department and its health services [52]. With a successful campaign which included IRS with insecticides, malaria was eliminated from the country by 1963 [52]. Consequent to malaria elimination, during the first POR stage, lax vector control measures resulted in increased mosquito densities in previously malaria-endemic regions. In 1990, due to an economic recession, the frequency of vector control was drastically reduced and a year later, the country was without available insecticides [52]. An established case detection system was still maintained at this time, but frequent surveys of the population were limited due to a reduction in transport and fuel facilities. Yet, in the face of these issues, the country maintained its malaria-free status up until 1993 notwithstanding malaria cases being imported into the country, due mainly to dedicated health care service providers [52].

However, in 1994, due to an internal conflict over the mountainous region of Karabakh, the intrinsic potential for malaria increased due to thousands of people being displaced and arriving into the country, and the uncontrolled mobility of the military [28]. As expected, the first indigenous case of malaria was detected in 1994 and most imported malaria cases detected (91.8%) were due to the refugees seeking asylum in the country from conflict zones. Appropriate treatment was not provided to those malaria patients due to the unavailability of primaquine in the country. Consequently, in 1996, health care services recorded a total of 347 cases of malaria, of which 149 were indigenous [52].

In 1998, Armenia established a comprehensive Roll Back Malaria programme to eliminate recently re-emerged malaria and to control the spread of the disease with financial assistance from WHO, governments of Norway and Italy, together with the technical assistance of WHO and the Red Cross [52]. Annual IRS with cyfluthrin was carried out. Bioinsecticide, *Bacillus thuringiensis israelensis* was also used for vector control, and mosquito larval growth in water bodies was controlled by introducing fish of *Gambusia* spp. [52]. A solid PCD system was emplaced district-wise, where diagnostic laboratories screened blood smears for malaria using microscopy; positive cases were notified at the central level to the Republican Centre of Hygiene and Epidemiological Surveillance in Yerevan, which acted as the national malaria reference centre. Subsequently, the positive cases were immediately (in less than 3 days) hospitalized at least for 5 days and treated patients after discharge were followed up by a physician [52]. The Armenian anti-malarial campaign was supported by the Armenian government, United Armenian Fund of the USA, WHO/Europe, United Nations Children’s Fund, the International Federation of the Red Cross and Red Crescent Societies [53].

Successful implementation of its new Roll Back Malaria programme as well as adequate funding resulted in Armenia earning its malaria-free certification again in 2011 [22]. Importantly, the country did not report any indigenous cases of malaria for a long-duration, in the course of many obstacles such as an internal conflict and lack of funding for primaquine and insecticides. This may be attributed to low intrinsic potential for malaria in the country due to its socioeconomic conditions, in addition to a dedicated health service.

Upon receiving malaria-free certification in 2011 the government of Armenia established a national programme for POR [54]. Major intentions were to adapt an epidemiological surveillance system to match with POR, establish a cross-border cooperation policy, health system consolidation for POR, spreading public awareness on POR and medical hygiene, recruitment of new staff and capacity-building in both new and existing staff for malaria prevention, establishing preventive measurements/activities for high risk groups such as frequent travellers to malaria-endemic regions and military personnel and assimilation of POR under the emergency programme activities [54]. The experience of re-establishment of malaria into Armenia after more than 30 years of interruption showed the importance of sustained surveillance for POR.

### Sri Lanka

Sri Lankan malaria history goes back to AD1300 and to the period of Dutch colonization (1658–1815) where malaria-like diseases were reported [55, 56]. Sri Lanka (then Ceylon) suffered a major epidemic of malaria in 1935 that recorded morbidity of over 1.5 million and mortality of 80, 000 [57]. However, by 1947 the country was implementing robust IRS island-wide which definitively controlled the disease [58]. As a result, in 1959, the country transitioned towards a malaria elimination campaign using the GMEP’s funding and strategy, which was made up of two key methods; intensive IRS and PCD. The campaign was tremendously successful where only 17 cases of malaria were documented by 1963, including 11 imported cases [58]. The country moved to a pre-elimination phase in 1964. The successes of the anti-malaria campaign caused overconfidence; DDT spraying was discontinued to prevent resistance and causing extensive under-reported cases. Consequently, in 1967 a *P. vivax* outbreak in two foci, resulted in a disastrous malaria epidemic in Sri Lanka from 1967–1968. The main reasons for this resurgence was that complacency had set in with IRS being scaled back, PCD programmes were not ratified, curtailing parasitic and vector surveillance, rainfall patterns, and reducing financial support due to the elation of the success [55, 59, 60].

DDT spray teams were reintroduced to cease the spread of the disease, but due to rampant vector resistance, DDT was ineffective. The malaria epidemic coincided with the discontinuation of the GMEP which was the major source of funding for malaria control in the island [58].

Sri Lanka received its first grant for malaria control from the Global fund to fight AIDS, Tuberculosis, and Malaria (GFATM) in 2002 [61]. Malaria prevalence in Sri Lanka declined again from 1999 and reached zero indigenous transmission by 2013 due to strong commitment to the rigorous malaria elimination programme. Microscopy or rapid diagnostic tests (RDTs) for rapid detection and confirmation of infections, parasitological surveillance along with monitoring and evaluation (both PCD and ACD), increasing annual blood smear examination rate, continuous elimination attempts in conflict areas regardless of 30 years of civil war (1983 to 2009), entomological surveillance as part of epidemic forecasting and vector management, use of IRS and ITN, and treatment with chloroquine and primaquine greatly contributed towards this success. Additionally, the economic development of the country vastly improved the probability of achieving the POR phase. Historically, malaria was predominantly a disease of the rural areas, where the mud houses with thatched roofs were good breeding grounds for the vector [62]. With the economic development of the country, these breeding grounds disappeared over time. In addition, development in communication and infrastructure over the past two decades led mobile malaria health care services to access remote areas of the country with ease.

Sri Lanka received malaria-free certification in 2016 and subsequently entered the POR phase. Being a country with high intrinsic potential for re-establishment due to its tropical climate and developing economy, POR of malaria remains a challenge [63]. Moreover, as the receptivity of the main vector *Anopheles culicifacies* remains high during the POR phase and high vulnerability of the population due to imported malaria cases being reported, Sri Lanka is at constant risk of malaria re-establishment [64]. This is further complicated with the reporting of *Anopheles stephensi*, the vector of urban malaria predominant in Southern India, from the Mannar district in Sri Lanka [65]. Therefore, the Anti-malaria campaign (AMC) of Sri Lanka is committed to prevent a repetition of malaria history of the island, and to continue to work with the same ardour and scrutiny to prevent re-introduction of the disease [64]. POR interventions are/were funded by the government of Sri Lanka (to the AMC through the Ministry of Health, and through provincial ministries for provincial POR activities) and through GFATM collaborations with the AMC (2016–2018) for POR [66]. WHO has also extended their support towards Sri Lanka continuously to keep malaria free through capacity building [66].

Essential operations that are being continued to keep the country free from malaria in the POR phase are, (i). Vector control (IRS, ITN, reduce stagnant water pools), (ii). Educating health personnel and the public on the risk of POR and on importation of malaria, and (iii). strong surveillance methods for the treatment of imported cases [56]. Sri Lanka has implemented the 1, 2, 3 approach where case notification, case investigation and responsive action was initiated in days 1, 2 and 3 from the date of detection further improving the WHO recommended 1,3,7 approach of China [61]. In Sri Lanka ACD (Microscopy is used mainly while RDTs are also used as a supplementary tool) is currently in use for screening high-risk groups and PCD to screen individuals with malaria-like symptoms visiting medical facilities post-elimination; however, a recent study revealed that maintaining awareness of the disease among physicians would be critical to effectively use PCD for detection of imported cases in the POR status [29]. Moreover, the AMC Sri-Lanka has further highlighted the need of a vigilant case surveillance and notification system, a high-risk group surveillance system, entomological surveillance, monitoring of insecticide susceptibility, pharmacological vigilance for anti-malarial medicines, and a rapid response team for each region to act quickly when a malaria case is reported under POR [66].

A major difficulty for POR in a tropical country such as Sri Lanka is the competitive demand for health care resources. Currently, dengue is widespread in the country with 51,659 cases reported in 2018 and 17,848 by March 2020 [67]. Therefore, in the aftermath of receiving malaria free certification from the WHO, as expected, political commitment and financial resources are directed to more demanding health care needs such as dengue. Importantly, Regional Malaria Officers of the AMC are also responsible for dengue control activities in their districts. The AMC is however working towards acquiring resources for the sustainable existence of a malaria POR programme.

### South Korea

The history of malaria outbreaks in Korea draws back into the twelfth and thirteenth centuries [68]. Later in 1913, 1328 and 147 cases of malaria were reported in Korean civilians and Japanese soldiers, respectively [69, 70]. Available information on the prevalence of malaria in South Korea reported that malaria was endemic and prevalent during the second world war (1941–1945), post-independence (1945–1950) and the Korean war (1950–1953) [69]. However, the incidence had increased remarkably during the Korean war [69]. In order to combat malaria, the government of Korea and WHO jointly established the National Malaria Eradication Services (NMES) in 1959. NMES consisted of spleen surveys to examine people with substantial spleen enlargement, mass blood surveys to examine *P. vivax* slide positivity in people, ACD that identify fever patients in assigned areas by periodic visits and administer antimalarial drugs (chloroquine) and PCD. In PCD, blood smears of all fever patients were sent to NMES laboratories and chloroquine treatment was provided. In addition, spraying of DDT in all indoor areas followed by PCD and mosquito surveys in DDT treated areas was also implicated [69]. However, the results of ACD, PCD, and DDT residual spraying revealed that PCD single-handedly would effectively control malaria in Korea [69]. Due to the continuous and strenuous efforts of NMES, malaria was declared eliminated in South Korea in 1979 [71]. However, in 1993 malaria remerged at the Demilitarized Zone (DMZ) bordering South Korea and North Korea [72]. The only plausible explanation for this is the dispersal of sporozoite-infected mosquitoes to South Korea from North Korea [73, 74]. “Insufficient anti-malarial efforts by North Korea” could also be the reason for resurgence along with epidemiological factors, such as wind velocity, temperature, and humidity [75]. Insecticide resistance and genetic diversity could be considered another probable reason as a previous study reported insecticide resistance in 70% of the mosquitoes in the DMZ [76, 77]. Malaria incidence increased rapidly after the first emergence in 1993 that peaked in 2000. An outbreak of *P. vivax* malaria was reported in 1998 with 2100 malaria cases.

Unavailability of an outbreak response plan, limited personal expertise in malaria programme management and disease diagnosis, limited capacity to improve vector control strategies lead to a nationwide spread of malaria reporting 601,013 cases between 1999 to 2001 [78]. Thus, in 2002 mass primaquine preventive treatment (MPPT) was initiated by the Ministry of Public Health with the assistance of WHO, and implication of MPPT significantly reduced the malaria burden in South Korea [78]. Large-scale chemoprophylaxis programmes decreased the malaria cases by 2012 [79, 80]. With 445 (2013), 638 (2014), 699 (2015), 681 (2016), 515 (2017), 501 (2018) cases from 2013–2018, fluctuations in annual incidence of malaria was reported since then, yet a declining trend is visible overall [1, 80]. Funded entirely by the national government, the researchers, academics, military, and the South Korean government are engaged in rapid patient discovery and treatment processes, and vector control plans entering the malaria pre- elimination phase [80].

For South Korea to reach elimination, it is critical to establish a cross border cooperation with North Korea, consider re-introduction of active case detection, as employed in the 1960s and 1970s, have a more intensive and systematic surveillance system with access to all information on malaria cases, epidemiological and entomological data, human movement, parasite genotyping [75, 80]. Yet, unavailability of sustained financing for malaria elimination and characteristics of the two *P. vivax* strains with relatively distinct incubation periods in North Korea can decelerate the South Korean elimination efforts indirectly. In 2018, the South Korean government established a 5-year action plan, fully funded by the national government to achieve elimination certification [1]. Improving public sector cooperation, implementing vector control at the DMZ and cross border cooperation are the critical challenges to be faced [1].

### India

India, with 2.6 million lesser reported cases of malaria in 2018 than that of 2017 still accounts for 4% of the global malaria burden and 47% of the *P. vivax* malaria cases globally [1]. India’s attempts to eliminate malaria has been challenging due to population movement across states/countries as a country sharing large international borders, large population size, shortage of skilled human resources, insecticide resistance, lack of robust monitoring and evaluation systems, lack of integration with private sector, lack of sustained financial and political commitment and hard to reach endemic areas [81].

Owing to its tropical nature, India is a country that is invariably affected by malaria. Achieving independence in 1947 freed the country to start laying the foundations for its own programmes against malaria. In 1953, the National Malaria Control Programme was launched with the aid of the WHO GMEP programme and it worked tenaciously to fight malaria, using a strategy based on constant IRS, prophylaxis, and therapy [82]. The programme was phenomenally successful, reducing the morbidity of malaria from 75 million cases in 1952 to a mere 100, 000 cases in 1965 [82]. The general speculation was that the country was well on its way to achieving malaria elimination. However, with the misconception of malaria being established only in rural areas, limited control strategies established in cities lead to the resurgence of malaria in ten cities, in 1965 [82]. Even though the countrywide incidence rates of malaria had declined, the major factors responsible for the resurgence of malaria in urban areas were, (i) increasing development of rural and urban areas that resulted in harbouring suitable conditions for mosquito breeding such as construction activities and irrigation for farmlands [82], and (ii) *Anopheles stephensi,* a relatively harmless vector mosquito that had adapted to urban environments, breeding in overhead tanks, cisterns, and wells [82]. This resurgence was progressively observed across the country. Several factors that contributed additionally to the countrywide spread of malaria were, (a) the Indo-Pakistan war, which caused the efficiency of the programme to decline due to hardships in accessing the conflict zones and, a large portion of the governmental budget allocated for the defence budget, (b) overconfidence of the government that malaria can be controlled due to the previously successful control programmes, which then led to complacency, (c) funding shortages as the GMEP of the WHO was discontinued, and d) emergence of both insecticide resistant vector mosquitoes and drug resistant parasites [39, 82]. Controlling the malaria outbreaks in urban areas was challenging due to the shortage of DDT, as a consequence of fund reductions after the country had successfully reached 100,000 cases [28]. As a result, large areas that were under the pre-elimination phase were reverted to malaria prevalent areas and the health care service infrastructure was not adequate or well established to perform vigorous surveillance in these areas. Malaria in the urban areas dominated and began diffusing into the rural areas [39]. The resurgence of malaria throughout India caused morbidity of malaria to increase from 0.1 million to 6.4 million from 1966 to 1976. A modified plan of operation was introduced in 1977, but failed and subsequently put forward again in the mid-1980s and yet again in 1995, none of which achieved success to any further extent [82].

Currently, malaria control strategies in India includes vector control through IRS and insecticide-treated nets, fish-based larval control methods, modern malaria surveillance using digital technology, routine vector surveillance and receiving community involvement in health education [83]. Microscopy and/or RDTs are used in ACD and at present, the Karnataka state of India is following the ‘1-3-7′ strategy to detect and treat every single case of malaria during the elimination phase [83]. Moreover, the Government of India launched the National Framework for Malaria Elimination (NFME, 2016–2030) to stop indigenous transmission, POR, and to receive malaria-free certification by 2030 [81, 84]. NFME has planned to complete its activities in four categories, (i) Category 3 (intensified control phase), (ii) Category 2 (pre-elimination phase), (iii) Category 1 (elimination phase) and (iv) Category 0 (POR phase). The main interventions of India’s POR programme are the detection of any re-introduced case of malaria and notification, determining the underlying causes of resumed local transmission and applying rapid curative and preventive measures [84]. Besides, policy planning, monitoring and evaluation, surveillance, stratification, quality assurance, intersectoral collaboration, cross-border collaborations, capacity building, and research are applied to all four phases of NFME activities [84]. Moreover, the National Strategic Plan (NSP)- 2017–2022 was established in 2017 by the Directorate of National Vector Borne Disease Control Programme (NVBDCP), the central nodal agency for the prevention and control of vector borne diseases in India, to target only the low and moderate transmission regions initially, where high burden districts will be focused after 2022 [81]. NSP control strategies include early case detection and prompt treatment, chemical and biological control of vectors, environmental management and community awareness, and ensuring personal protection against mosquito bites [81]. The national government mainly funds the Indian malaria elimination campaign where recently the government has increased the funding by more than 25% and increased the support as a donor to the GFATM [1, 85].

## Discussion

It is imperative to recognize the importance of utilizing all available resources, to implement strong campaigns for surveillance and control of malaria to prevent re-establishment. Particularly at this stage, as malaria incidence rates in the world are declining dramatically, a majority of malaria-endemic countries may gain pre-elimination or elimination status shortly. The way forward for these countries requires a well planned gold standard POR programme to be in position, the elements of which may inlcude predictive models, vector control, research, diagnostics, vaccines, funds, and political will.

### Predictive models

In addition to strong control programmes, utilizing predictive models has proven to be useful to ascertain potential outbreak risks and malaria transmission foci. A comprehensive study performed in Iran produced a carefully calibrated predictive model that incorporated different sets of predictive variables that can be easily calculated in the field, to predict malaria re-establishment 8 weeks in advance [86]. Previous groups have also introduced statistical methods for estimating the infection rate, prevalence and for eliminating reporting delays in surveillance systems [87, 88].

### Vector control

WHO has reported ITNs and IRS as the two core interventions for malaria vector control. These interventions also include larval control through environmental modification (draining and filling), usage of larvicides, biological control using fish, bacterial toxins; *Bacillus thuringiensis *var. *israelensis* (Bti), and usage of fungal varieties (e.g., *Laegenidium giganteum*) or mermithid nematodes (e.g., *Romanomermis culicivorax*) [89]. Other measures include fogging and area sprays, introduction of sterile male mosquitoes and genetic manipulation of vectors; a relatively novel method [89]. CRISPR/Cas9 (Clustered regularly interspaced short palindromic repeats/CRISPR associated protein 9) mediated gene editing is a potential game-changer, bringing in permanent disease control by creating gene drives [90, 91]. CRISPR/Cas 9 technology gene editing was used for population suppression in *Anopheles gambiae*, the mosquito vector for malaria in the African continent, by effecting female sterility [92] and to suppresses *Plasmodium* infection *in An. gambiae*, by inactivating the fibrinogen-related protein 1 (FREP1) [93]. Furthermore, CRISPR/Cas 9 technology was used to produce *An. stephensi* that are resistance to *Plasmodium sp*. [94].

### Research

Researching and incorporating new techniques, such as predictive models, genetic engineering, m-health initiatives, and most importantly, malaria vaccines into POR programmes is critical for the evolution of malaria prevention programmes. Additional research must be performed in various countries, with said research being easily accessible. The volume of information regarding POR programmes from countries that have reached elimination is limited. Many articles from Mauritius were found to be documented in French, or on location hard copy archives at the Ministry of Health in Mauritius. English articles from Armenia were limited with a majority being documented in Armenian.

Advancements in technology can be applied to enhance malaria surveillance activities. A study conducted in Papua New Guinea used an m-health initiative to strengthen malaria surveillance in a 184-health facility, multi-province, project aimed at strengthening the National Health Information System (NHIS) in a country with fragmented malaria surveillance, which is moving towards pre-elimination [95]. This study demonstrated that using mobile technologies and GIS in the capture and reporting of NHIS data in Papua New Guinea provides timely, high quality, geo-coded, case-based malaria data required for malaria elimination. Such data enables all malaria control stakeholders to access the data with a programme that is simple to use. With this information, the data can be mapped to a health facility or village level so that transmission foci can be visualized, and responses targeted. The health system strengthening approach of integrating malaria information management into the eNHIS optimizes sustainability and provides enormous flexibility to cater for future malaria programme requirements [95].

### Diagnostics

Malaria diagnosis requires highly sensitive, reliable and easy-to-perform methods including microscopy, rapid diagnosis tests (RDT), polymerase chain reaction (PCR), or a combination of these methods [100]. Being a low cost technique, microscopy of Giemsa-stained thick and thin blood smears considered as the reference standard [100], is routinely used to screen for malaria in both malaria prevalent countries and countries under the POR stage [96]. However, the accuracy of the test substantially vary dependent on the microscopists’ training with a range of 5–100 parasites/µl [97]. Use of microscopy in combination with another diagnostic technique whenever possible would be ideal in a POR setting.

RDTs, are also used widely in malaria endemic areas irrespective of its inability to detect very low levels of parasitaemia (below 100–200 parasites/μl) [98]. To overcome the sensitivity issues related to detecting low-density malaria, Ultra-sensitive Malaria Ag *P. falciparum* RDT (uRDT) was developed and assessed recently in the field, and was identified with high sensitivity [99, 100]. *Plasmodium falciparum* histidine-rich protein 2 (HRP2) antigen is reported to be commonly used for RDT due to its high expression level and multi-epitope avidity [99, 100]. The HRP2-based uRDT has shown promising results in identifying high prevalent communities and has performed better than conventional RDT and microscopy at low parasitaemias [99, 100].

Use of nucleic acid amplification techniques (NATs), such as Polymerase chain reaction (PCR) and Loop-mediated isothermal amplification (LAMP) is recommended in a POR setting [101, 102]. PCR is the most routinely utilized NAT, used for its high sensitivity and accuracy in diagnosing the presence of malaria parasites in blood [103]. A single parasite in a blood sample can be detected by PCR, being the most sensitive technique over the techniques cited above [104]. The need for trained professionals to perform the convoluted technical procedure and to handle expensive equipment limits PCR being used for malaria diagnosis in any other setting less than an elimination/POR setting. In a POR setting, the number of indigenous cases of malaria will be zero, with only a few possible imported cases. This provides an ideal setting for the use of pooled PCR and positive results in a pool can then lead to screening of individual samples in the pool. In a POR setting with no outbreak risks, the cost efficiency is better than performing individual RDTs in the same setting, an additional advantage of requiring a lower load of work [103]. LAMP is a novel, highly specific and sensitive NAT, which is faster than PCR, identified in 2001 [102, 105]. LAMP has been used to identify human *Plasmodium* species and has the potential to be used in diagnosing both traveller screening and population-screening rendering it an ideal tool for a POR setting [105, 106]. Moreover, unlike the most highly specific molecular diagnostic tools that require an electric supply, LAMP functions on its own exothermic reaction proving it an ideal tool for field use [105, 106]. Furthermore, a recent meta-analysis study based on 66 studies (based on both symptomatic or asymptomatic patients), confirmed that the LAMP method is robust for diagnosing malaria when compared to RDT and microscopy [97]. However, in a developing country with non-availability or limited access to modern diagnostic tools, microscopy performed by an experienced microscopist can be used as a sound POR diagnostic tool.

### Vaccines

The most viable option for preventing malaria could be a malaria vaccine. To date, there is no commercially available vaccine to fight against malaria [107]. However, malaria vaccine development has not impeded progress, since its beginnings in the 1930s, to develop a functional, efficient, mass-producible vaccine against one of the deadliest parasites known to man [108].

Several prospective vaccines with a spectrum of approaches towards preventing malaria have been identified with the majority of them targeting a single stage of the parasite’s life cycle. Vaccines against the parasite pre-erythrocytic stage have shown success [108]. The pre-erythrocytic *P. falciparum* candidate vaccine, RTS,S—a hybrid protein particle formulated in a multicomponent adjuvant, the first trials of which were published in 1997, was successful in phase III efficacy trials and favourably reviewed by the European Medicines Agency and WHO. Currently, RTS,S has been introduced into national pilot implementation programmes, which marks the first human anti-parasite vaccine to pass regulatory scrutiny [107, 108]. Testing has increased of other pre-erythrocytic candidates that target sporozoite- or liver-stage parasites, mainly the whole sporozoite vaccines. Inadequate human efficacy of asexual blood-stage vaccine candidates, that targeted to limit blood-stage parasite growth, resulted in a plummeted interest in these. Transmission-blocking vaccines, that kill sexual stage parasites in the vector mosquito, advanced to field trials over the last decade. Notably, the first generation of placental malaria vaccines that clear sequestering parasites in the placenta entered the clinic over the last decade [109, 110]. Novel antigen discovery, human monoclonal antibodies, structural vaccinology, and improved platforms promise to expand on RTS,S and improve existing vaccine candidates [110]. Multi-component vaccines that targets more than one life cycle stage or combination of two pre-existing partially effective vaccine candidates would be more effective as a vaccine candidate.

Funding has remained a salient factor worldwide during the fight against malaria, regardless of the stage that the country is in (pre-elimination/ elimination/ POR stage). This is attributed as one of the largest burdens, especially in low Gross Domestic Product (GDP) economies [28]. Developing countries must rely on obtained funds to continue to perform these expensive programmes, especially in states of elimination/pre-elimination. International and domestic funding for malaria control and elimination totalled US$ 3.1 billion in 2017 [38]. Although this represents a significant increase since 2005, when the total funding was US$ 960 million, it still needs to be increased to US$ 6.6 billion by 2020 to achieve global malaria targets by 2030 [3, 38].

A well founded, strong public health infrastructure supported with adequate, trained field staff for contact tracing and testing were common factors for the success of both malaria elimination and the current rigorous programme operational for the malaria prevention of re-establishment in Sri Lanka [109]. Yet the national malaria control programme, the Anti-Malaria Campaign (AMC) of Sri Lanka, is daunted with many challenges during the current POR, as malaria no longer is a major public health threat; declining funding for malaria from the Global Fund, waning political interest and a rising disinterest toward malaria among local health workers due mainly to other health issues, i.e. dengue fever and non-communicable diseases, being current national health priorities [110]. In this milieu, it may be prudent to presume that incorporating POR measures into the elimination-planning programme will be crucial to sensitize both the medical community and the government on the need to maintain resources and focussed attention to the risk of malaria re-establishment in a country.

## Conclusion

Re-establishment of malaria after its elimination has caused enormous loss to a country’s economy and to the lives of people; therefore, prevention of re-establishment (POR) of malaria is crucial. Countries that have eliminated malaria recently such as Sri Lanka should learn from their own mistakes and by those made by other countries as discussed in the review, to sustain a malaria-free status. POR should be achieved by focusing on reducing the levels of receptivity and vulnerability of the region as it determines the probability of re-establishment. Therefore, each country should develop a tailor-made POR programme especially depending on the epidemiological and entomological surveillance data in addition to the generalized recommendations provided by the WHO.

## Data Availability

Not applicable.

## Abbreviations

ACD: Proactive/active case detection
ACT: Artemisinin-based combination therapy
AMC: Anti-Malaria Campaign
CRISPR/Cas9: Clustered regularly interspaced short palindromic repeats/CRISPR associated protein 9
DDT: Dichlorodiphenyltrichloroethane
DMZ: Demilitarized Zone
DNA: Deoxyribonucleic acid
GDP: Gross Domestic Production
GMEP: Global Malaria Eradication Programme
HRP2: Histidine rich protein 2
IRS: Indoor residual spraying
IVM: Integrated Vector Management
LAMP: Loop mediated isothermal amplification
MPPT: Mass primaquine preventive treatment
NAT: Nucleic acid amplification technique
NFME: National Framework for Malaria Elimination
NHIS: National Health Information System
PCD: Passive case detection
PCR: Polymerase chain reaction
POR: Prevention of re-establishment
R_0_: Basic reproduction number of malaria
RACD: Reactive/activated passive case detection
RBM: Roll Back Malaria
RDT: Rapid Diagnostic Test
USAID: United States Agency for International Development
WHO: World Health Organization
